# How resistant is anammox biofilm against antibiotics: A special insight into anammox response towards fluoroquinolones

**DOI:** 10.1016/j.heliyon.2024.e41339

**Published:** 2024-12-18

**Authors:** Faysal-Al Mamun, Rohit Kumar, Kelvin Ugochukwu Anwuta, Sovik Das, Madis Jaagura, Koit Herodes, Tetyana Kyrpel, Agnieszka Fiszka Borzyszkowska, Anna Zielińska-Jurek, Zane Vincevica-Gaile, Juris Burlakovs, Andrey E. Krauklis, Mohamad Nor Azra, Md Salauddin, Jiexi Zhong, Taavo Tenno, Kai Bester, Ivar Zekker

**Affiliations:** a Institute of Chemistry, University of Tartu, 14a Ravila St., 50411, Tartu, Estonia; b Indian Institute of Technology Delhi, Hauz Khas, New Delhi, 110 016, India; c Institute of Genomics, University of Tartu, Riia 23b, Tartu, 51010, Estonia; d Institute of Chemistry, Chair of Analytics, University of Tartu, 14a Ravila St., 50411, Tartu, Estonia; e Department of Environmental Toxicology, Faculty of Health Sciences, Medical University of Gdansk, Dębowa Str. 23A, 80-204, Gdansk, Poland; f Department of Process Engineering and Chemical Technology, Gdansk University of Technology, Narutowicza 11/12, 80-233, Gdansk, Poland; g Department of Environmental Science, University of Latvia, Jelgavas Street 1, LV-1004, Riga, Latvia; h Faculty of Civil and Mechanical Engineering, Riga Technical University, LV-1048, Riga, Latvia; i ASEMlab – Laboratory of Advanced and Sustainable Engineering Materials, Department of Manufacturing and Civil Engineering, NTNU – Norwegian University of Science and Technology, 2815, Gjøvik, Norway; j Institute of Climate Adaptation and Marine Biotechnology, Universiti Malaysia Terengganu, 21030, Kuala Nerus, Terengganu, Malaysia; k UCD Dooge Centre for Water Resources Research, School of Civil Engineering, University College Dublin, Ireland; l Department of Environmental Science, Aarhus University, Frederiksborgvej 399, Roskilde, 4000, Denmark; m WATEC – Centre for Water Technology, Aarhus University, Ny Munkegade 120, Aarhus, 8000, Denmark

**Keywords:** Nitrogen removal, Moving bed biofilm reactor, Anammox, Pharmaceutically active compounds, Adsorption

## Abstract

Elevated concentrations of pharmaceutically active compounds (PhACs) in the water bodies are posing a serious threat to the aquatic microbiota and other organisms. In this context, anaerobic ammonium oxidizing (anammox) bacteria carry a great potential to degrade PhACs through their innate metabolic pathways. This study investigates the influence of short-term exposure to lower and higher concentrations (0.8 mg L^−1^, 0.06 mg L^−1^, respectively) of antibiotics on the anammox process under distinct operational conditions (starvation/non-starvation) in moving bed biofilm reactor (MBBR). During batch operations that lasted for up to 6 h, the total nitrogen removal efficiency (TNRE) and total nitrogen conversion rate (TNCR) reached a maximum of 93 ± 5 % and 6.97 ± 1.30 mg N g^−1^ TSS d^−1^, respectively. Evidently, at higher PhAC levels, the anammox process was active, and up to 75 % PhAC removal efficiency was obtained within 6 h of the batch cycle. Most importantly, the anammox biofilm effectively eliminated the PhACs compounds, i.e., ciprofloxacin (CIP), ofloxacin (OFL), and norfloxacin (NOR) present at higher (0.8 mg L^−1^) and lower (0.06 mg L^−1^) total PhACs (sum of CIP, NOR, OFL) concentrations. Furthermore, 16S rRNA sequencing analyses showed a mixture of nitrifying, denitrifying, and anammox bacterial commodities enriched on the carriers' surface with a high relative abundance of *Candidatus* Brocadia, primarily responsible for catalyzing the anammox process. This study showed the intricate relationship between PhAC concentrations, TNCR, and antibiotic elimination in the wastewater treatment, and the results obtained set up a new breakthrough in wastewater treatment. Future research should investigate the mechanisms that underlie the anammox biofilms' resistance to various types of PhACs and investigate the long-term stability and scalability of this process with real wastewater influents.

## Graphical abstract

**Figure:**
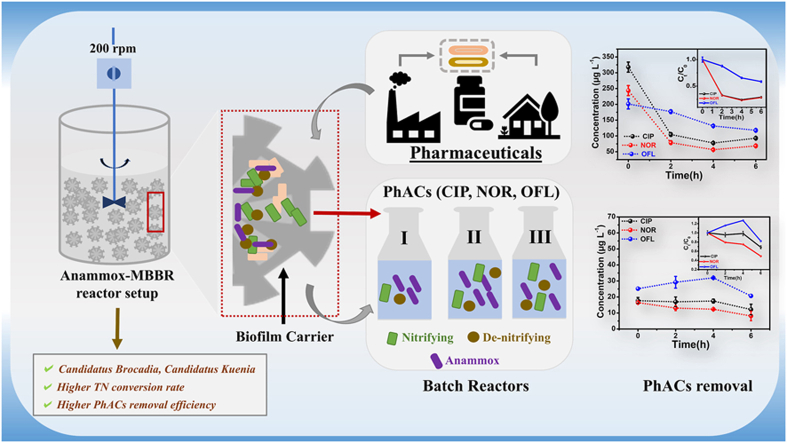

## 1 Introduction

The discharge quality standards of wastewater treatment plants (WWTPs) have become stringent due to the increased agricultural practices, fossil fuels consumption, and untreated household wastes, that drives the reactive nitrogen compounds and antibiotics into the water bodies and risks the aquatic flora and fauna. Nitrification and denitrification are commonly adopted by most of the WWTPs to remove nitrogen biologically in an economic and efficient manner, but at the same time the processes consist of some unavoidable drawbacks [[1], [2], [3], [4]]. Therefore, to avoid eutrophication emerging in the aquatic environment, anaerobic ammonium oxidizing (anammox) bacteria-assisted nitrogen removal has gained specific attention over the past few decades, as it offers an direct conversion of NH_4_^+^ to N_2_ under anoxic conditions using NH_4_^+^ as an electron donor and NO_2_^−^ as an electron acceptor [[5], [6], [7], [8]]. Several studies suggest that anammox process can also remove pharmaceutically active compounds (PhACs), such as those belonging to the fluoroquinolone group present in the waste streams. The toxicity level and degradation mechanism of these PhACs vary drastically even with a minimal changes in the operational conditions i.e. molecular structure and size, solubility, concentration, surface charge and medium's pH [9]. For instance, ciprofloxacin (CIP) is often recommended by experts against a wide array of bacterial infections, and its overuse in the last century have considerable increased its concentration in the water bodies, surpassing the national regulatory standards issued by USEPA for safe WWTPs effluent discharge into the environment [[10], [11], [12], [13]]. Apart from CIP, a high concentration of other antibiotics i.e. amoxycillin (AMX), penicillin G, norfloxacin (NOR) have been detected in a diverse range of aquatic environments including bank filtrates, wastewater, surface water and even groundwater, which demands an immediate scientific attention and effective solution. The conventional activated sludge (CAS) process is one of the most widely used biological treatment methods in WWTPs due to its efficacy in simultaneously removing both- organic matter and nutrients. The process relies on the activity of a diverse aerobic microbial community that degrades the organic pollutants present in the wastewater and consequently metabolize the harmful antibiotic compounds by simply breaking them into simpler forms, which are often less harmful and does not require any further treatment before discharging into environment. However, it is very challenging to remove fluoroquinolone antibiotics by the CAS, as it leads to the accumulation of the PhACs in residual waste sludge [14]. Industrial WWTPs mostly use the adsorption process for PhAC residues removal that primarily involves the physico-chemical interactions between the antibiotic molecules and the surface of the activated carbon. During physical adsorption process, weak van der Waals interactions, pore filling/size-selective adsorption and π-π interactions occur while chemical adsorption involves the formation of surface chemical bonds between the adsorbate and adsorbent either by equally sharing the electrons or forming a coordinate bond by electron transfer through ion/ligand exchange. The applicability of this method primarily depends on the type of adsorbent material used (activated carbon, carbon nanotubes, ion-exchange resins etc.) as well as on the structure and reactivity of the antibiotics present in the wastewater. It is often observed that operational conditions, such as adsorbent dose, pH, and temperature significantly affect the adsorption kinetics of PhACs [15]. Adsorption capacity of CIP onto sludge could be up to 6.3 mg per kg dry matter, and adsorption has been considered the dominant removal method for PhACs elimination in the CAS processes [10], but could end up with desorption and inhibitions occurring. Biodegradation efficiencies of CIP were reported to be 3.53–17.15 % in the CAS process [10]. Undoubtedly, adsorption process is a cost-effective and efficient method to remove a variety of pollutants from the industrial wastewater, but indeed demands a high adsorbent dosage and operational energy to obtain favorable results. Regeneration of the adsorbent material poses a serious challenge to WWTPs, which often require post-treatment of the adsorbent material before reuse. Therefore, enhancements of the material morphology to boost the selective adsorption of antibiotics from the waste streams is imperative. Advanced oxidation processes show great performance in antibiotic degradation, but their low mineralization capacity is a serious issue as they can generate many oxidation intermediates and products [16]. The unsuspected toxicity of some degradation products is one of the disadvantages of this process. Moreover, they might bring antibiotic resistance genes (ARGs) in microbes, especially pathogenic bacteria [17]. Moving bed biofilm reactors (MBBR) have emerged as a promising candidate for biological treatment of antibiotic compounds because of its robust design, high removal rates per unit of biomass, and smaller ecological footprint [18]. MBBRs rely on the bacterial biofilms that are grown on small plastic carriers, which are suspended and mixed in the reactor continuously and maintaining a homogeneous nutrients availability for the bacterial commodities is required. In many report, researchers have found almost 100 % removal efficiency for CIP in the MBBR system [18,19]. PhAC removal can be improved by an enhanced nitrification process by increasing aerobic cycle lengths compared to the anoxic ones, most likely due to increasing metabolic biodegradation by ammonium oxidizing bacteria (AOB) [20]. Heterotrophic (HET) bacteria present in the biofilm may also accelerate the metabolic biodegradation of PhACs due to their organotrophic nature [21]. PhACs at pH < 7 i.e. ibuprofen, ketoprofen, naproxen, diclofenac, clofibric acid, mefenamic acid, and gemfibrozil have shown enhanced removal during the nitrification process occurring in the MBBR system [22]. It should be considered that anammox process cannot remove some specific group of PhACs (i.e. erythromycin and tetracycline) when present at higher concentrations (0.1–4 mg L^−1^) [23]. A previous study on PhAC removal in the MBBR found that 55 % of CIP was removed under starvation conditions maintained by a lack of ammonium supply, whereas 50 % of CIP was adsorbed in the dead biomass [24]. It has been shown that NOR had a severe impact on several wastewater treatment systems [25]. The effects of NOR at higher concentrations (1 mg L^−1^) on anammox activity was investigated for 30 days, and it showed that NOR significantly interfered with microbial activity due to toxicity [26]. The degradation rate is faster during the aerobic starvation phase compared to that achieved during the anaerobic starvation phase, which means that when exposed to aerobic starvation conditions, anammox bacteria would rapidly lose PhAC degradation functionality [14,27]. Anammox and partial nitrification processes conducted in a MBBR under high antibiotic concentrations have achieved the highest antibiotic removal rate of 295 μmoL g^−1^ L^−1^ h^−1^ [28]. A recent study conducted showed that for CIP, a removal efficiency of 53 ± 1 % during the 12-h operational period was achieved [24]. It was concluded that 44 % of the removal was attributed to adsorption and only 9 % to biodegradation. Another study described the removal of NOR, which achieved a remarkable 99 % removal efficiency using an anammox microbial consortium in an aerated biofilter [29]. Ilmenite-biochar composites have been investigated as an adsorbent material for removing ofloxacin (OFL) [30]. A removal efficiency of 99 % was found, and the synergistic effect of pore filling, hydrogen bonding, and π-π interactions was the primary driving force for achieving the remarkable removal efficiency. There are a limited number of reports that describe the anammox microbial metabolism to remove fluoroquinolone compounds, especially when one or more PhACs (CIP, NOR, and OFL) are present together in waste streams, as the complex chemistry involved makes it challenging to target their effective removal [30].

Therefore, this study provides an special insights into the inhibiting characteristics of three different PhACs (CIP, NOR, and OFL) on the anammox activity. The long and short-term exposure of PhACs with broad concentration range (0.06 mg L^−1^ to 0.8 mg L^−1^) was investigated to understand the adaptive and concentration-dependent response of anammox under varying testing conditions (anoxic and aerobic/anoxic). Three PhACs were selected because they exist in actual wastewater streams and are reported to pose hazardous impacts on the environment. There are few studies that have investigated the antibiotics concentration-dependent response of anammox biofilm at trace concentration (0.001 mg L^−1^). Therefore, from this work, we have systematically evaluated the anammox performance at starvation and at higher loadings of PhACs at non-starvation phases. The kinetics of CIP, NOR, and OFL were analyzed by zero and first-order kinetic models. Findings from this study pave the way for addressing future wastewater treatment needs, particularly for the PhACs industries' WWTPs.

## 2 Materials and methods

### 2.1 Chemicals

Ciprofloxacin (CIP, C_17_H_18_FN_3_O_3_ ≥ 98 %, Merck), norfloxacin (NOR, C_16_H_18_FN_3_O_3_ ≥98 %, Merck), ofloxacin (OFL, C_18_H_20_FN_3_O_4_ >99 %, Merck), sodium nitrite (≥97 %, Sigma-Aldrich), ammonium chloride (99.9 %, Sigma-Aldrich), magnesium sulfate (≥95 %, Sigma-Aldrich), calcium chloride (97 %, Alfa Aesar), iron chloride hexahydrate (97 %, Sigma-Aldrich) were of analytical grade and used without further purification. If not stated otherwise, all the reagent solutions were prepared using ultra-pure Milli-Q water.

### 2.2 MBBR setup and operation

Anammox bacteria were cultivated in a cylindrical plexiglass MBBR reactor (height = 52 cm, diameter = 25 cm) having a working volume of 20 L. The dissolved oxygen (DO) concentration (<1 mg L^−1^) in the MBBR was maintained by providing 45 min of aeration followed by a short non-aeration period lasting for 15 min. The DO analyzer (Elke Sensor, Estonia) connected with the MBBR monitored the DO level in the system, and a mechanical stirrer attached from the top maintained the homogeneous availability of the nutrients for the anammox bacteria. About 10,000 Kaldnes K1-shaped biofilm carriers (specific surface area = 800 m^2^ m^−3^) were used to support the growth of bacterial biofilm. The influent pH was found in the range of 6–8.5 and was manually adjusted with 1 M HCl or 1 M NaOH whenever needed. The hydraulic retention time (HRT) was set at 3 days. Reject wastewater collected from the Tartu wastewater treatment plant consisting of minerals and trace elements was used as the *inoculum* source for the cultivation of anammox bacteria. Besides, reject wastewater also contains a variety of antibiotic compounds, e.g., CIP, erythromycin (ERY), levofloxacin (LEV), NOR, OFL, sulfamethoxazole (SMX), and trimethoprim (TIM), at a concentration >20 μg L^−1^. Therefore, to mimic the real environmental conditions, no additional PhACs were added to the MBBR reactor. However, during the batch tests, different PhAC concentrations ranging from 60 to 800 μg L^−1^ were supplemented to bacteria and studied to understand the concentration-dependent response of anammox bacterial species. The MBBR was operated in starvation and non-starvation conditions to examine the anammox's long term performance in nitrogen removal and antibiotic degradation under low to minimal and normal feeding conditions. During the non-starvation phase, the MBBR was continuously supplied with a mixture of NH_4_^+^-N coming from reject water to support the growth of anammox. Besides, during the starvation phase, no additional source of NH_4_^+^-N and NO_2_^−^-N was provided. The current research aimed to assess the resilience and adaptability of the anammox bacteria under nutrient-limited conditions to enhance their capability for PhAC removal. The specific starvation and non-starvation control conditions for both phases were crucial in evaluating the anammox bacteria's efficiency and stability under varying operational scenarios.

### 2.3 Sample collection and N-removal analysis in MBBR

The concentration of all the N-species (NO_2_^−^-N, NO_3_^−^-N, and NH_4_^+^-N) was estimated using a Hach Lange DR 2800 spectrophotometer. The experimental results of the batch procedure adopted for the measurements are summarized in the supplementary material (Fig. S1). The inorganic compounds total nitrogen (TN, in mg N L^−1^) in the influent (TN_in._) and effluent (TN_eff._) sample was estimated using Eq. (1). At first, the influent and effluent samples were centrifuged at 4500 rpm for 15 min using Allegra® X-15R centrifuge and the supernatant was used for TN analysis. Moreover, the pH (of the influent and effluent) and temperature of the MBBR were frequently monitored during operation. Eq. (2) and Eq. (3) were used to estimate the total nitrogen removal rate (TNRR) and total nitrogen removal efficiency (TNRE):

(1) TN_inorganic_ = NH_4_^+^ + NO_2_^−^ + NO_3_^−^

(2) $$TNRR=\frac{\left(Influent\;TN-Effluent\;TN\right)}{Daily\;Loading\;Q\;\left(m^{3}d^{-1}\right)}$$

(3) $$TNRE=\frac{\left(Influent\;TN-Effluent\;TN\right)}{Influent\;TN}\times 100\%$$

### 2.4 Batch studies for PhACs removal

The three-neck plexiglass glass reactors with working volume of 1 L were used to study the PhACs removal tests in bath mode. Two different PhACs concentrations (high: 0.80 mg L^−1^ and low: 0.06 mg L^−1^) were selected to evaluate the potential role of anammox in degrading the antibiotics at different concentrations and conditions. The polyethylene cylindrical-shaped biofilm-containing carriers (∼200) were transferred from the pre-acclimitised MBBR to the batch reactors. The batch tests were performed for a duration of 6 h at 25 ± 1 °C in two different conditions: 1) strictly anoxic and 2) in a combined aerobic/anoxic environment (fluctuated for 1 h each). If not stated otherwise, the already prepared stock solutions of CIP (50.05 mg L^−1^), NOR (40.09 mg L^−1^), and OFL (20.09 mg L^−1^) were used to reach the desired PhACs concentration for batch tests. Before tests, the biofilm carriers were gently washed 3 times in Milli-Q water not to detach the biofilm from the carrier surface.

The synthetic wastewater solution (pH ∼ 7.5–8.0) consisting of 2 mL NaNO₂, 2 mL NH₄Cl, 0.4 g H₂CO₃, 1 mL of phosphate buffer, 1 mL MgSO₄ × 7H₂O, 1 mL CaCl₂, 1 mL FeCl₃ × 6H₂O, 1 mL alkaline trace element solution, and 1 mL of acidic micro-nutrient solution was added to the batch reactors. The concentration of micro- and macro-elements was selected based on previously reported work [31]. The initial concentration of NH_4_^+^-N and NO_2_^−_^N was 95 ± 6.90 mg N L^−1^ and 35 ± 5.89 mg N L^−1^, respectively. Samples were collected at a fixed time interval after every 2 h. To ensure accurate and reproducible results, experiments were conducted in three replicates.

### 2.5 Biomass dry weight

The dry weight of the biomass was determined using a meticulous process, commencing with weighing 20 biofilm-containing carriers, which were subsequently rinsed with deionized water and subjected to a 24-h drying process at 105 °C in an oven. After the initial drying, the carriers were weighed, followed by the biomass removal using concentrated chromic acid. The biofilm carriers were further washed with deionized water and dried at 105 °C for 24 h. The final step involved calculating the dry weight of the bio-carriers by subtracting the weight difference between the carriers with and without the biomass.

#### 2.5.1 PhACs concentration determination

The high-performance liquid chromatography-mass spectrometry **(**HPLC-MS) was used to estimate the PhACs' concentration. At first, 5 mL of the PhAC sample was weighed and transferred to a 250 mL beaker containing 100 mL of Milli-Q water and named as solution A. Afterward, 1 ± 0.1 mg L^−1^ sodium ethylenediaminetetraacetate (Na_2_EDTA) was added to solution A, and the pH was adjusted to ∼4 using formic acid (HCOОH). Solid-phase extraction (SPE) is then performed using preconditioned cartridges (HLB 6 cm^3^, 500 mg LP, 60 μm) with methanol, Milli Q water, and 10 mM Na_2_EDTA. Prepared samples were loaded onto the cartridges at a flow rate of 3 mL min^−1^. The sample-containing flasks were rinsed with 3 mL of 5 mM HCOOH solution (pH = 4). The elution of the residues from the cartridge was achieved using 12 mL of methanol (MeOH), and the weight of MeOH was recorded. Subsequently, 0.3–1 mL of the MeOH extract was evaporated in a vacuum centrifuge at 40 °C and 70 mbar pressure. The resulting sample was reconstituted into 0.5–1 mL of solvent (0.1 % formic acid (FA): methanol = 9:1). A possible 10-fold dilution involves combining 0.1 mL of the MeOH extract with 0.9 mL of a 0.1 % FA solution. The final step involves HPLC-MS analysis, utilizing a calibration solution consisting of 0.1 % FA: MeOH = 9:1.

#### 2.5.2 16S rRNA pyrosequencing and microscopic analysis of the biofilms

In environmental conditions, the anammox bacteria co-exists with other group of bacterial species i.e. nitrifiers and denitrifiers. Therefore, to understand the specific abundance of anammox and other related microbial communities, 16S rRNA sequencing analysis was performed. For the analysis, 20 biofilm carriers (each containing about 3 mg of bacterial biomass) were transferred from the MBBR into a 20 mL falcon tube. The mixture was then shaken vigorously to remove the attached biomass from the carriers. The biofilm bacterial composition was tested with next-generation sequencing (NGS), and DNA extraction was performed using the reported method stated by Ref. [32]. The V4 hypervariable region of the 16S rRNA was amplified using F515 5′-GTGCCAGCMGCCGCGGTAA-3′ and R806 5′-GGACTACHVGGGTWTCTAAT-3′ primers [33] and sequenced on the iSeq 100 platform, as previously described in Ref. [34]. The BION-meta program was used to perform taxonomic profiling on the sequence data (https://github.com/nielsl/mcdonald-et-al) according to the author's instructions by applying the protocol described earlier in Ref. [2]. The micrographs of the biofilm were recorded on a light microscope (Invitrogen, USA) with a phase contrast set at different magnifications (eyepiece 10 × , objective lenses 40 × and 60 × ). Micrographs were taken by dual digital eyepiece camera head (Leica, USA) at different resolutions, ranging from 20 μm to 300 μm.

### 2.6 Statistical analysis and kinetic modelling

A one-way ANOVA Tukey testing was adopted to detect F (degrees of freedom) and P values, where P values < 0.05 were considered statistically significant. To understand the kinetics of the reaction, the experimental data was fitted with zero-order and first-order models and the regression coefficient (R^2^) was estimated from the linear regression and used to assess the fitness of experimental data to specific reaction kinetics.

## 3 Results and discussion

### 3.1 MBBR performance

**Fig. 1:**
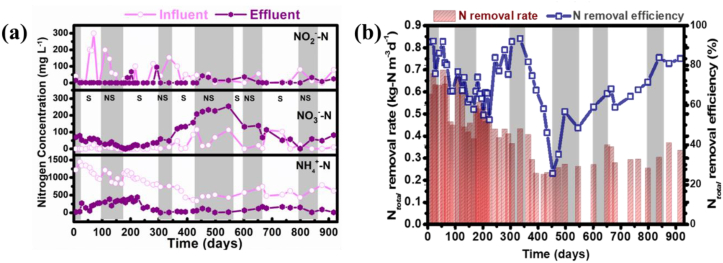
(a) Changes in NO_2_^−^-N, NO_3_^−^-N, and NH_4_^+^-N concentrations and (b) correlation between total nitrogen removal rate (TNRR) and total nitrogen removal efficiency (TNRE) achieved in the MBBR during the operational period of 900 days including non-starvation (NS, grey) and starvation (S, white) phases.

The performance of the pre-acclimitised MBBR was evaluated in the starvation and non-starvation phases. During starving conditions, essential nutrients i.e., NH_4_^+^-N and NO_2_^−^-N that support the anammox growth were absent whereas, during the non-starvation phase there was provided a continuous supply of NH_4_^+^-N and NO_2_^−^-N, allowing the successful conversion of these compounds into N_2_ by anammox bacteria [35]. The concentration of each nitrogen species, i.e., NH_4_^+^-N, NO_2_^−^-N, and NO_3_^−^-N was monitored monthly, and these components changes are shown in Fig. 1a. It can be inferred that the NH_4_^+^-N concentration in the effluent decreased significantly from 760 mg N L^−1^ to 12 mg N L^−1^ after 130 days of operation. Also, apart from a decrease in the NH_4_^+^-N concentration, a subsequent increase in the NO_3_^−^-N concentration was observed, which is the characteristic by-product of the anammox metabolic pathway. After the first 200 days of operation, a considerable decrease in NO_2_^−^-N concentration (from 154 mg N L^−1^ to 0.92 mg N L^−1^) was observed. The TNRE reached a maximum of 83 ± 8 %, during starvation period while significantly lower TNRE was obtained during the non-starvation period. The lowest TNREs of 26 ± 9 % and 57 ± 6 % were found at around 350–450 days in the starvation and non-starvation periods, respectively. This can be attributed to the lack of nutrients supplied during the starvation period and the slow overcoming of the non-starving period from the starvation period. Due to the change in reaction stoichiometry during the anammox process, a shift in effluent's pH was anticipated. Therefore, a correlation of pH change between influent and effluent samples can be seen in Fig. S3. Evidently, there was a minimal change in the influent/effluent pH during the starvation phase. In contrast, a more significant decrease in the effluent pH was encountered during the non-starvation phase, which was attributed to the aerobic oxidation of NH_4_^+^ and rapid production of nitrate by anammox bacteria, which contributes to the drop in pH of the effluent.

Table 1 summarizes the operational performance parameters of the MBBR. The influent of the MBBR mainly consisted of NH_4_^+^-N and NO_2_^−^-N. The TNRR was lower in the first days of reactor operation and reached a high value at the end of the starvation and non-starvation periods. During the non-starvation phase, the continuous supply of NH_4_^+^-N and NO_2_^−^-N supported the successful conversion of ammonium to N_2_ by anammox bacteria (maximum TNRE of about 93 ± 5 %). However, during starvation phases, the lack of nutrients most likely led to a higher efficiency in NH_4_^+^-N consumption as the bacteria targeted available nutrients for maintaining metabolism and cell structure. These results evidenced that a well-functioning anammox bacterial culture was successfully obtained after the enrichment process, which was further assessed to evaluate its potential in eliminating the PhACs.

**Table 1:** Operational performance of the MBBR during the starvation (S) and non-starvation (NS) phases within the 917 days of operation. Inf-wastewater influent, eff.-wastewater effluent.

Phase (S/NS)	Time (d)	NH_4_^+^-N inf. (mg N L^−1^)	TN inf. (mg N L^−1^)	TNLR (kg N m^−^³d^−1^)	NH_4_^+^-N eff. (mg N L^−1^)	TN eff. (mg N L^−1^)	TNRR (kg N m^−^³d^−1^)
NS	7–30	1334.1 ± 112.89	1355.6 ± 80.30	0.63 ± 0.046	107.93 ± 21.86	181.56 ± 7.32	0.54 ± 0.070
S	31–100	1139 ± 129.19	1262.96 ± 52.26	0.59 ± 0.10	193.68 ± 49.25	248.06 ± 33.28	0.47 ± 0.130
NS	110–182	1009 ± 129.24	1075.94 ± 92.56	0.50 ± 0.09	320.10 ± 41.98	340.23 ± 31.58	0.34 ± 0.090
S	189–300	969.10 ± 89.45	1015.17 ± 108.34	0.46 ± 0.05	275.02 ± 32.35	315.06 ± 56.32	0.32 ± 0.047
NS	300–350	740.29 ± 15.58	1001.18 ± 58.47	0.40 ± 0.02	18.03 ± 1.89	94.10 ± 1.16	0.35 ± 0.040
S	355–430	475.50 ± 57.28	533.6 ± 65.15	0.24 ± 0.03	32.98 ± 6.35	173.32 ± 25.14	0.16 ± 0.045
NS	435–600	442.26 ± 28.78	546.25 ± 29.68	0.25 ± 0.01	60.88 ± 8.63	297.62 ± 26.26	0.11 ± 0.037
S	650–800	581.16 ± 74.32	652.03 ± 81.41	0.305 ± 0.04	147.50 ± 18.54	221.36 ± 22.62	0.20 ± 0.030
S	840–917	683 ± 73.15	717.15 ± 57.76	0.336 ± 0.03	39.10 ± 4.35	124.76 ± 15.84	0.27 ± 0.022

### 3.2 Effect of PhACs on TN removal efficiency

**Fig. 2:**
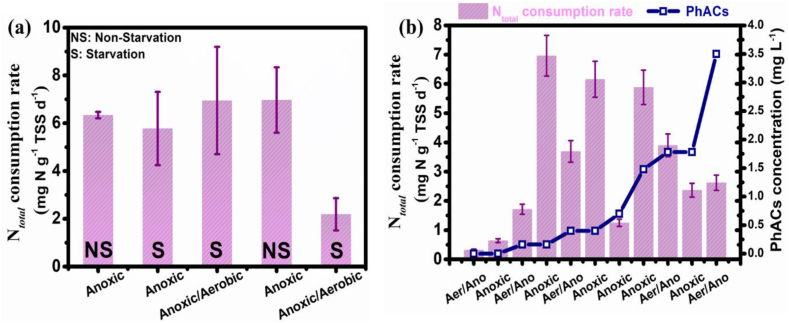
(a) Batch test comparison without PhACs presence under different anoxic non-starvation (Ano-NS), anoxic starvation (Ano- S), and aerobic/anoxic starvation (Aer/Ano- S) conditions compared with PhACs concentration in anoxic non-starvation (Ano- NS-Phar) and aerobic/anoxic starvation (Aerobic/Anoxic (S- Phar)) conditions. The first 3 data points are without PhACs presence, and the last 2 data points are with PhACs applied. (b) Batch test at different concentrations of PhACs in aerobic and anoxic (Aer/Ano) and anoxic conditions. Error bars show the standard deviation between triplicate tests.

To evaluate the TN removal potential of anammox bacteria in presence of different PhAC compounds (CIP, OFL, NOR), it was investigated and compared in terms of TN consumption rates (TNCR) at both-starving (S) and non-starving (NS) conditions. All the nitrogen forms, including NO_2_^−^-N, NO_3_^−^-N, and NH_4_^+^-N, which contribute to TNCR, were shown during tests at fixed time intervals operated in combined aerobic/anoxic and only anoxic conditions. These conditions are abbreviated as “aer/ano” and “ano”, respectively. The tests were performed for a duration of 103 days, including a control test (without added PhACs), which was held for the first 36 days of the test duration. Fig. 2 shows that under aer/ano and S conditions, referred to as the control experiment, the TNCR of 6.95 ± 2.50 mg N g^−1^ TSS d^−1^ was observed in the batch cycle (without the addition of PhACs).

**Fig. 3:**
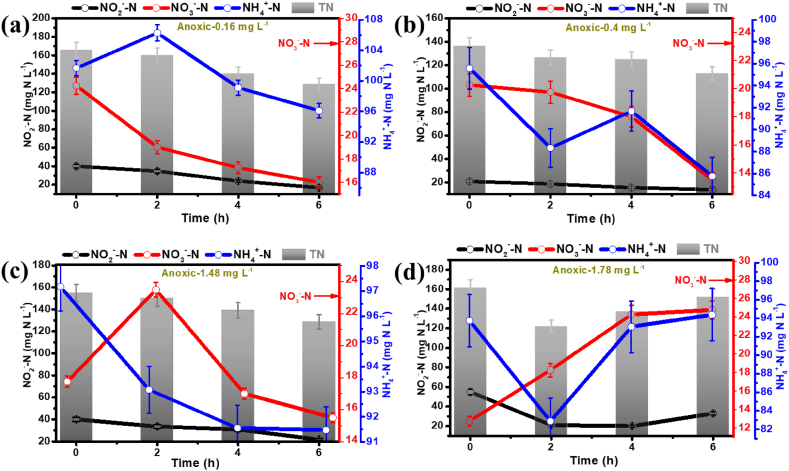
Changes in nitrogen species (NH_4_^+^-N, NO_2_^−^-N, and NO_3_^−^-N) concentration with time under anoxic conditions and in the presence of different PhACs initial concentrations [(a) 0.16 mg L^−1^, (b) 0.40 mg L^−1^, (c) 1.48 mg L^−1^, and (d) 1.78 mg L^−1^].

Similarly, under anoxic NS and S conditions tested without any PhACs presence, the TNCRs of 6.33 ± 0.13 mg N g^−1^ TSS d^−1^ and 5.77 ± 1.53 mg N g^−1^ TSS d^−1^, respectively, were achieved. The TNCR in the presence of PhACs (mixture of CIP, NOR, and OFL) with an initial concentration of 0.16 mg L^−1^ was about 6.97 ± 1.37 mg N g^−1^ TSS d^−1^, very close to the value observed in aerobic/anoxic and starvation conditions when no PhACs concentration was present. However, in the aer/ano tests in S conditions, the lowest TNCR value of 2.19 ± 0.67 mg N g^−1^ TSS d^−1^ was observed, which was considerably (but statistically, insignificantly) lower compared to control test (one-way ANOVA *F* = 0.930, *p* = 0.058). This shows that in aer/ano conditions, the anammox activity was inhibited due to the interference of PhAC compounds. Recent studies showed that CIP at a concentration of ≥6453 ng L^−1^, obstructs microbial growth in activated sludge [36]. Therefore, the tests were performed at various initial concentrations of PhACs ranging from 0.16 mg L^−1^ to 3.50 mg L^−1^ under both-ano and aer/ano conditions. At a low PhAC concentration of 0.40 mg L^−1^, the highest TNCR of about 6.96 ± 1.36 mg N g^−1^ TSS d^−1^ was observed under anoxic and NS conditions. However, a reverse trend was observed for higher PhAC concentrations (3.50 mg L^−1^), where TNCR of 2.62 ± 0.15 mg N g^−1^ TSS d^−1^ was observed in aer/ano and NS conditions. Control tests compared the process performance in both-anoxic and aerobic/anoxic conditions, revealing a distinct advantage for anoxic conditions, as shown in Fig. 2b. In addition, the NH_4_^+^-N consumption rate increased from 0.14 mg N g^−1^ TSS d^−1^ to 3.19 mg N g^−1^ TSS d^−1^ when the PhAC concentrations were increased from 1.78 mg L^−1^ to 3.50 mg L^−1^ (Fig. S1). The trend suggested a very significant correlation: higher PhAC concentrations correspond to increased consumption of NH_4_^+^-N levels, proving that anammox process is still active in the presence of increased PhACs levels. This relationship is further supported by Fig. 3, illustrating that anammox bacteria removed higher PhAC concentrations more efficiently within the initial 2 h when higher PhACs concentrations were present. The analysis of TNCR with and without PhACs supplied indicated that the anammox process remains effective in both scenarios, with a limited adverse impact of PhACs. Several factors may have contributed to this observation, including aerobic/anoxic conditions, PhAC concentrations, and the potential development of PhAC-resistance by anammox bacteria due to prior exposure to wastewater containing PhACs. Anammox bacteria are known for their resilience and ability to adapt to adverse conditions, including high antibiotic concentrations. When exposed to antibiotics, the nitritating, denitrifying and anammox bacteria can enhance their metabolic activity and express specific genes that help in detoxifying and degrading the antibiotics, leading to improved overall performance in nitrogen removal. Also, antibiotics can trigger co-metabolic pathways, changing the bacteria's core metabolic activities to incorporate antibiotic breakdown. This can result in an overall increase in metabolic rate and efficiency, leading to a better TNCRs during antibiotic-stress conditions.

Studies also showed that NOR (a fluoroquinolone antibiotic and a bacteriolytic activity broad-spectrum antibiotic), works by blocking a specific DNA gyrase enzyme responsible for bacterial DNA synthesis and repairment [37]. The irreversible inhibition threshold for NOR on the anammox bacteria ranges from 50 to 100 mg L^−1^, as reported by previous studies [37]. However, the influence of reactor type and influent source remains a subject of interest, as the limited diversity in reactor design and predominantly synthetic wastewater source used in previous studies may not fully clarify the complex interaction between antibiotics and anammox bacteria activity. This trend suggests an inverse relationship between PhAC concentration, TNCR and the removal of PhAC compounds, highlighting a potential trade-off between removal processes within the system. Fu et al. (2021) also mentioned in their work that investigations are needed to establish a comprehensive understanding of the anammox response to fluoroquinolone antibiotics removal [38].

Simultaneously, as can be seen from Fig. 3, a noticeable increase in the NO_3_^−^-N concentration was observed at lower PhAC levels. This increase confirmed the successful conversion of ammonium and nitrite to N_2_ and nitrate by the anammox process and that the PhAC compounds were not inhibiting the anammox bacterial activity. Further investigation of this correlation might provide more insights into the interactions between microbial communities under anoxic conditions.

**Fig. 4:**
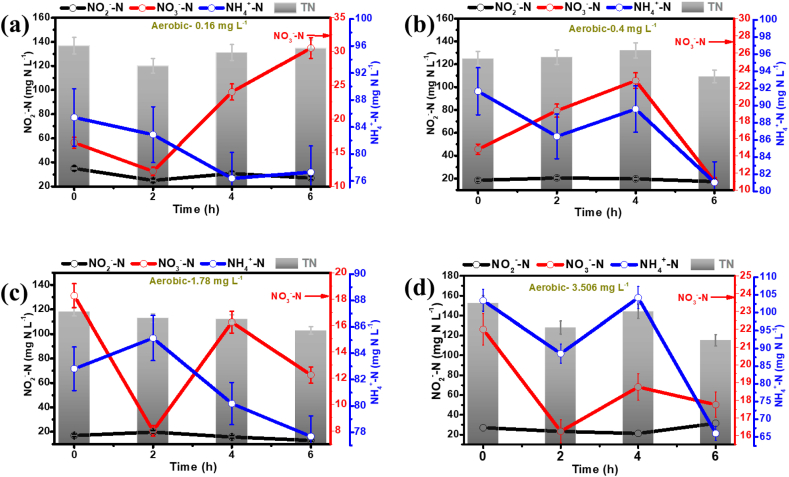
Changes in nitrogen species (NH_4_^+^-N, NO_2_^−^-N, and NO_3_^−^-N) concentration with time under aerobic/anoxic (1 h/1 h) conditions and in the presence of different PhACs inlet concentrations [(a) 0.16 mg L^−1^, (b) 0.40 mg L^−1^, (c) 1.78 mg L^−1^, and (d) 3.51 mg L^−1^].

Fig. 4 showed that under aerobic/anoxic conditions, the drop in NH_4_^+^-N concentration utilized by anammox bacteria was less significant than drop achieved under anoxic condition in case for all the PhAC concentrations applied. This suggests that the metabolic pathways observed for microbial populations involved in nitrogen removal were different in nitrogen removal contribution between anoxic conditions. In the batch test with PhAC compounds applied at a concentration of 1.48 mg L^−1^ under aer/ano condition, within the first 2 h, there was an increase in NH_4_^+^-N concentration, and a sharp fall in NO_3_^−^-N levels occurred. However, at lower concentrations of PhAC compounds (0.16 mg L^−1^), there was less NH_4_^+^-N removal and higher NO_3_^−^-N production observed for different concentrations of PhAC supplied during the batch tests run. This relationship showed nitrogen removal dependence on PhAC compounds in aer/ano conditions with enhanced ammonium removal at low PhAC concentrations.

### 3.3 Short-term PhACs removal tests and kinetics

**Fig. 5:**
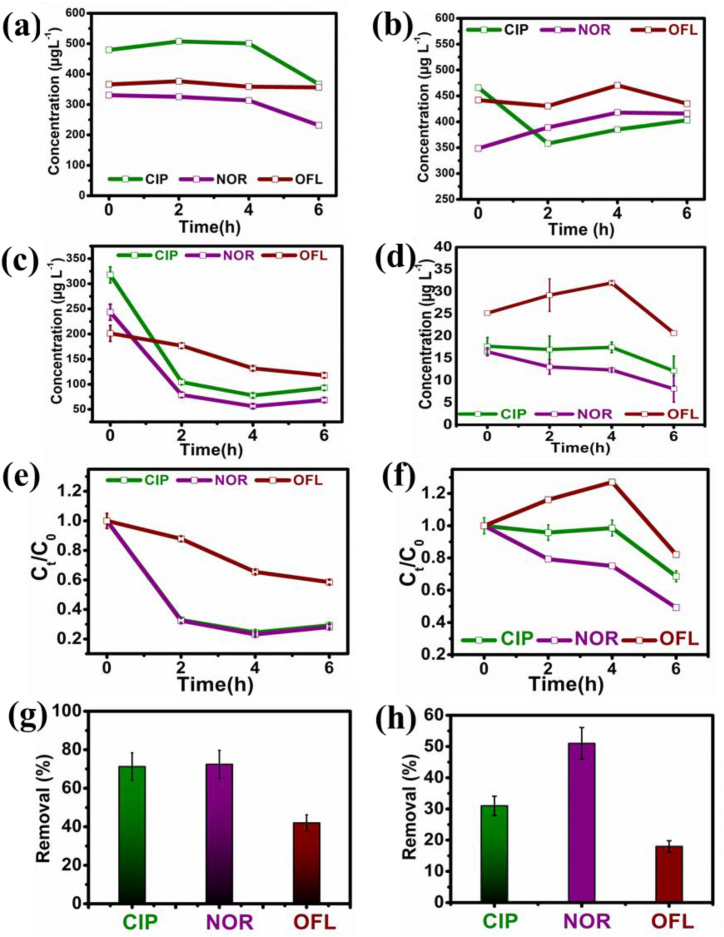
(a) Control (abiotic) tests in anoxic condition, (b) Control (abiotic) test in aer/ano condition, (c) Time-dependent higher PhACs concentrations removal tests, (d) Lower PhACs concentrations drop with time (non-starvation anoxic), (e) (C_t_)/initial (C_o_) PhACs changes with time (t) for higher concentration of PhACs (f) C_t_/C_o_ vs. t, for lower concentration of PhACs degradation (g) Removal % of the PhACs (CIP, NOR, OFL) at higher concentrations (h) Removal % of the PhACs (CIP, NOR, OFL) at lower concentrations.

A short-term (6 h) PhAC removal studies were conducted to primarily provide initial insights into the removal efficiencies and determine the immediate response of the microbial community in the presence of antibiotics (Fig. 5). Along with nitrogen removal tests, the removal of PhACs with varied initial concentrations was studied. Initially, at 0 h, the concentrations of CIP, NOR, and OFL concentrations were 317.80 ± 32.87 μg L^−1^, 243.50 ± 19.48 μg L^−1^, and 201.30 ± 18.17 μg L^−1^, respectively (Fig. 5). Throughout the testing period of 6 h, the concentrations of CIP, NOR, and OFL were steadily decreased, showing lowered concentrations of 92.95 ± 4.65 μg L^−1^, 68.55 ± 4.78 μg L^−1^, and 117.75 ± 4.89 μg L^−1^ for CIP, NOR, and OFL respectively, as shown in Fig. 5c. The removal efficiencies for CIP, NOR and OFL were found to be 71.27 ± 6.71 %, 72.42 ± 7.92 %, and 38.97 ± 2.59 %, respectively, as shown in Fig. 5g. As per the reported studies, the piperazinyl ring of fluoroquinolones takes part in various substitution and decomposition reactions of PhACs. De-ethylation and deamination reactions are primarily ones involved in breaking the piperazinyl ring [24]. Deamination, catalysed by deaminases in the presence of AOBs, and the creation of hydroxyl radicals from hydroxylamine during ammonium oxidation may speed up the degradation of CIP and NOR [39]. Research showed that the piperazine ring present in the PhACs is mainly responsible for antibacterial activities that can convert PhACs to less toxic products when the piperazine ring breakdown occurs, which mainly indicates the ability of the nitrifying bacteria to eliminate the antimicrobial activities of the PhACs [25]. A steep decrease in the concentrations of CIP and NOR was observed during the first 2 h for higher concentrations (Fig. 5c). Thereafter, the removal rate of PhACs was constant, which indicates the saturation of adsorption sites on the bacterial cell surface, or it can be attributed to the inhibition of anammox process. During the abiotic anoxic control experiments (shown in Fig. 5a and b), a slight decrease in the CIP and NOR concentrations was observed, whereas OFL was not removed at all. This implies that within anoxic conditions, without the presence of bacteria, only limited PhAC quantities may partially be degraded. Nevertheless, no PhAC degradation was seen in the aerobic/anoxic condition (shown in Fig. 5a and b). For the lower PhACs quantities supplied (0.06 mg L^−1^), the initial concentration for CIP was 17.7 ± 1.20 μg L^−1^, for NOR it was 16.45 ± 1.97 μg L^−1^, and for OFL it was 25.15 ± 2.10 μg L^−1^. After 6 h of degradation the concentrations were found to be 12.15 ± 0.67 μg L^−1^, 8.10 ± 0.85 μg L^−1^, and 20.65 ± 1.05 μg L^−1^, respectively. The PhACs removal efficiencies were found in the order of 31 ± 0.11 % for CIP, 51 ± 0.15 % for NOR, and 18 ± 0.25 % for OFL. It can be interpreted that during the initial 4 h of testing, a slight decrease in the PhAC concentrations was observed, where CIP and NOR followed a similar trend, but OFL showed no significant removal during the initial 4 h with a slight drop in concentration detected during the last 2 h. The lowest PhAC removal efficiency was observed for OFL measured at low initial OFL concentration involved in the test (18 μg L^−1^), which was 18.0 ± 0.25 % (Fig. 5d). The highest removal efficiency of 51 ± 0.15 % was observed for NOR (Fig. 5d). Consequently, it can be concluded that PhACs degradation, particularly CIP and NOR, appears to be condition-dependent, happening at higher efficiency under anoxic conditions, but not in the aerobic/anoxic conditions. Catabolic breakdown at high PhAC concentrations (0.1–1 mg L^−1^) may be possible since there is loose bacterial biomass present that may utilise antibiotics as carbon and energy source. These short-term batch experiments were employed to assess the interaction between anammox bacteria and specific antibiotics (CIP, NOR, OFL) under controlled low water retention conditions. Short-term batch experiments are useful for determining initial removal rates in real WWTP at high wastewater flow rates, to detect immediate effects of nitrogen removal, and assess potential toxicity of the compounds on the microbial community.

Apart from that fortuitous metabolic breakdown happening at lower applied PhAC concentration tests, the organisms continue to apply their normal primary metabolism depending on the biodegradable organic compounds of the wastewater and break down the antibiotics without involving new enzymes.

**Fig. 6:**
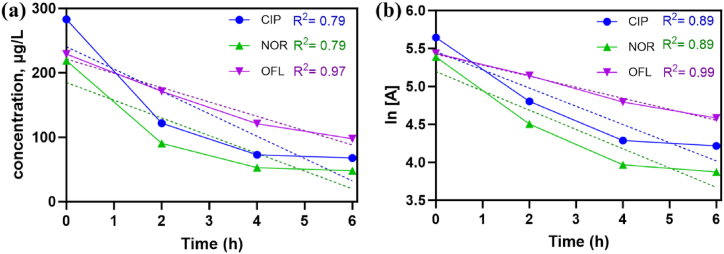
(a) Zero-order and (b) First-order kinetic model fitting for PhACs compounds degradation.

The zero-order and first-order fitting are shown in Fig. 6. For higher concentration, a high regression coefficient (R^2^) of 0.89 for NOR and CIP indicates that the PhACs degradation was well-fitted by the first-order kinetic model (Fig. 6b). This indicates that a first-order process can effectively define the rate of reaction for both- CIP and NOR degradation. For the compound OFL, R^2^ = 0.99 suggests a better fit of the first-order kinetic model to the experimental data. All three compounds indicate that first-order kinetics gives a good fit for explaining the decay of CIP, NOR, and OFL under the experimental conditions studied. The calculated kinetic constants (k) for CIP, NOR, and OFL were: 0.24 h^−1^, 0.25 h^−1^, and 0.14 h^−1^, respectively. The values obtained fall in a similar range as reported earlier for MBBR fluoroquinolone treatment [24]. Reports have suggested that the presence of ammonium ions considerably impacts PhAC removal rates. Authors have reported that a CIP removal % of about 55 ± 0.2 % was observed in the presence of ammonium, whereas the CIP removal % reached 84 ± 1 % when there was no ammonium present [24]. A particular adsorption experiment was carried out, and the respective details are summarized in (Table S1). It was observed that 14 ± 0.58 % of PhACs were accumulated into the loose biomass in case of CIP, while 5 ± 0.39 % and 12 ± 0.87 % of NOR and OFL compounds were adsorbed into the loose biomass, respectively. Previous literature suggested that adsorption was observed to contribute in up to 50 % of the CIP capture by dead biomass [24]. The removal rate deviated at lower concentrations of PhACs (below 10 μg L^−1^). This effect also corresponds to the Michaelis-Menten model kinetics, which results are shown to have a slowdown of PhACs breakdown at higher PhACs concentrations due to enzyme saturation [40].

Other factors, like anoxic and aerobic conditions presence, can also affect the degradation of PhACs by the anammox process. However, anammox bacteria have a long propagation time (>1 week), and they are extremely sensitive to their surroundings [41]. It was observed that the OFL removal efficiency is the lowest for both-at high and low initial PhAC concentrations, as shown in Fig. 5 (c) and 5(d). It can also be mentioned that OFL presence could decrease the diversity and richness of the microbial community represented in the reactor. The differences in removal % parameter values for different PhAC compounds may indicate microbial communities' preference for specific biofilm carrier structures, which requires further investigation.

### 3.4 Microbial community analysis

**Fig. 7:**
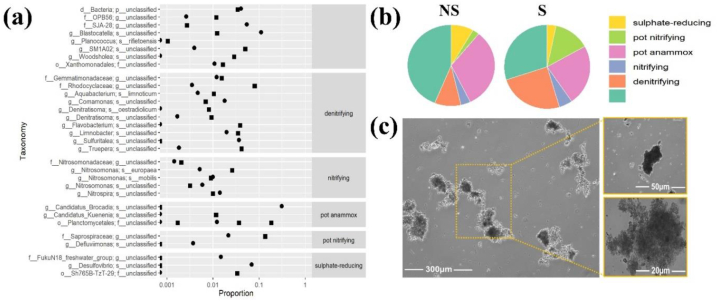
(a) Taxonomical profiles of nitrogen-removing bacterial species enriched during starvation (square) and non-starvation (circle) in the Anammox-MBBR biomass (b) Microbial community structure in phylum levels revealed by 16S rRNA gene amplicon sequencing in starvation and non-starvation phases (c) Micrographs of the biofilm of anammox bacteria recorded at different resolutions ranging from 20 μm to 300 μm.

The most prominent bacterial groups among the ammonium oxidizers/nitrifying bacteria, *Nitrosomonas s.* and *Nitrospira s.* were dominant during the starvation and non-starvation phases Fig. 7 (a). It also shows that throughout the non-starvation period, the *Candidatus Brocadia* genus was encountered at high proportions for the overall anammox community enriched during the experiments.

In contrast, it was observed that during the phase of starvation, the relative abundance of anammox bacteria species was reduced by about 5 % of the total anammox species abundance. This probably happened due to the absence of sufficient growth nutrients in the system during starvation periods. The microbial communities in the biofilm were exhibited at different levels, as shown in Fig. 7b, with *Candidatus Brocadia, Candidatus Kuenenia*, and *Planctomycetales* identified as the main represented strains of anammox bacteria. Interestingly, *Planctomycetes* were constantly present in starvation and non-starvation stages, compared to other bacteria species which were occasionally absent. 16S rRNA sequencing analysis showed a high abundance of the denitrification bacterial species *Rhodocyclaceae, Flavobacterium, and Truepera* in the MBBR reactor (Fig. 7). Several reports suggest that *Candidatus Kuenenia* is the most abundant anammox bacterium strain capable of removing nitrogen from the wastewater streams. So far, *Candidatus Kuenenia stuttgartiensis* and *Candidatus Brocadia anammoxidans* (freshwater species) and *Candidatus Scalindua sorokinii*, *Candidatus Scalindua wagneri*, and *Candidatus Scalindua brodae* (marine species) have been suggested as dominant anaerobic ammonia-oxidizing bacteria (AnAOB) [42].

Zhang et al. (2022) also identified *Candidatus Brocadia* in their study to be responsible to remove antibiotics from wastewater [43]. The same species were also identified in our current work. Due to the changes in biofilm structure under inhibitory circumstances, the *Candidatus Brocadia* population relative abundance decreased to 1.6 % in the biofilm and to 0.1 % in the sludge. The PR1 strain of *Achromobacter denitrificans* can degrade the sulfonamide class drugs [44]. OFL as a fluoroquinolone antibiotic, when treated with *Trametes versicolor*, could undergo 80 % degradation [45]. Fluoroquinolone antibiotics (CIP, OFL, NOR) get degraded by *Pseudoxanthomonas* in culture medium in the presence of carbon. Fig. 7b also highlights that during the non-starvation phase, anammox bacterial species dominated over other nitrifying and denitrifying bacterial species, suggesting their significant role in enhancing PhACs concentration removal, as shown in Fig. 6c. Remarkably, the presence of anammox bacterial species relative abundance did not significantly decrease during the non-starvation phases.

The microorganisms belonging to phylum *Proteobacteria, Firmicutes, Chloroflexi, Bacteroidetes, Thermotogae, Euryarchaeota, Elusmicrobia, Chlorobi, Spirochaetes, Synergistetes, and Actinobacteria* are important for stable performance of anaerobic digestor in terms of antibiotics removal. For instance, Liu et al., 2015, in a recent study, showed the existence of 18 different anaerobic bacterial species (*Longilinea, Georgenia, Desulforhabdus, Thauera, Desulfuromonas, and Arcobacter*) in the sewerage system [46]. Additionally, treated effluent may contain fecal bacteria from the genera *Bifidobacterium* and *Bacteroides* [47]. In this study, a minimal amount of *Longilinea arvoryzae, Thauera humireducens, Thauera terpenica, Thauera phenylacetica,* and *Desulfuromonas palmitatis, Bacteroides caccae, Bacteroides salanitronis* were detected and require additional in-situ studies to confirm their individual role for the degradation of PhACs.

The captured microscopic images of the anammox biofilm detected after 900 days of operation from the MBBR carrier's biomass are shown in Fig. 7c. The biofilm with different sizes and thicknesses can be seen as dark spots, which can be attributed to the extracellular polymeric matrix and to bacterial cell colonies. It can be concluded that different types of bacterial species are enriched on the carrier surface, which is also evident from the 16S rRNA analysis shown in Fig. 7a.

### 3.5 Possible anammox-assisted PhACs degradation mechanism

In the presence of antibiotics, a decrease in the anammox functional enzyme activity could be observed, which results in the deterioration of the anammox process performance. CIP is an antibiotic that particularly targets gyrase DNA, a enzyme responsible for catalyzing the ATP-dependent negative super-coiling of double-stranded closed-circular DNA during replication and transcription [48]. Recently, Wang et al. (2021) reported that the invasion of antibiotics in the anammox cells induces the production of reactive oxygen species (ROS), which damages the organism's DNA, proteins, and lipids [49]. The superoxide ion (O_2_^−^) is converted to peroxide (H_2_O_2_) by the anammox metabolism, which when combined with Fe^2+^ (from heme C), activates the process, and produced hydroxyl radicals (OH∗) induce cell damage [50].

A noticeable inhibiting effect of the antibiotics on microorganisms is more prominent at a concentration >1 mg L^−1^. Below this concentration, anammox bacteria can protect themselves by expressing antibiotic-resistant genes (ARGs) and maintaining the stability of nitrogen removal performance. This phenomenon can be observed when a higher PhACs concentration (>1 mg L^−1^) was present showing a steep decrease in antibiotic concentration observed during the initial 2 h, which is attributed to stronger adsorption of PhACs at the surface of the EPS matrix due to the presence of hydroxyl (OH^−^) and carboxyl (COO^−^) groups. However, due to the PhAC penetration into the first barrier provided by the EPS matrix, the anammox process starts to become inhibited, which, as a result, slows the PhAC degradation rate. This process was also reflected by the higher NH_4_^+^ removal during the initial 2 h of operation, which slowed down afterward showing a possible inhibition of the anammox process (Fig. 4). Moreover, based on the antibiotic class, different ARGs could be expressed by long-term MBBR reject water treatment (containing PhACs), as also demonstrated by varied removal rates present at different PhAC concentrations in our batch tests.

### 3.6 Applicability of the work

It is worth mentioning that antibiotics concentration is increasing significantly in the wastewater currently due to elevated use of them and hence, it demands an effective and sustainable solution to develop antibiotic-resistant treatment systems. Anammox process could show great potential and stable performance even when the PhAC concentrations reach a few mg L^−1^, which is often not detected in the real scenario. Also, conventional techniques have been unable to remove pathogens and other micropollutants, including antibiotics, antimicrobial resistant bacteria, and antimicrobial resistant genes [45,46,51]. Limited removal performance depended on the specific properties of the micropollutant, which was varied from lower to higher removal efficiencies. Therefore, this study presents anammox as an eco-friendly technology to rapidly treat antibiotic-rich wastewater and, more specifically, waste streams where CIP, OFL, and NOR PhACs are represented in a mixture. In addition, the present work illustrates the potential use of anammox under different operational conditions to effectively remove nitrogen as well as PhACs from the rejected wastewater representing N and PhACs at high levels. Also, this study confirmed that a robust and mature anammox bacterial biofilm is pivotal to sustain such a high-stress environment and harvesting a noteworthy output from the anammox communities. It can be emphasized that ARGs are secreted by the anammox bacteria though a protective mechanism against various antibiotics tested, which should be further explored to get a better understanding of the PhACs degradation mechanism involved. Therefore, it is necessary to explore anammox-based antibiotic treatment to find new insights into its mitigation and elimination mechanisms. The study showed insightful and promising fluoroquinolone PhACs removal results, but also has several new research questions that should be acknowledged:

The experiments were conducted under controlled laboratory conditions. Translating these findings to real-world applications in large-scale wastewater treatment plants may present challenges due to differences in scale, influent composition, and operational dynamics. The assessment of antibiotic removal was primarily conducted over short batch cycles (6 h). Longer-term studies could provide insights into the sustained performance and resilience of the anammox biofilm against fluctuating antibiotic concentrations and complex wastewater matrices. In this study authors utilized a specific type of biofilm reactor (MBBR) with a particular configuration. Other reactor designs or biofilm setups may exhibit different performance characteristics, necessitating comparative studies to optimize treatment efficiency. Addressing these shortcomings through further research and validation efforts will be essential for advancing the understanding and implementation of anammox-based technologies [51] in sustainable wastewater PhAC treatment practices.

Future research should focus on investigating the ideal conditions for anammox bacteria to thrive and interact with fluoroquinolone antibiotics. Longer experimental periods could yield more comprehensive insights into the long-term stability and efficiency of anammox biofilms in degrading PhAC compounds. Additionally, exploring the mechanisms underlying anammox bacteria's resistance to various PhACs and scaling up this process for real wastewater applications are critical steps toward developing more effective and sustainable wastewater treatment technologies. By addressing these aspects, we can enhance the robustness and applicability of anammox-based treatments in mitigating antibiotic pollution and improving water quality.

## 4 Conclusion

This study underscores the potential of the anammox process as a promising approach for the effective removal of nitrogen and fluoroquinolone antibiotics (CIP, OFL, NOR) from wastewater. The anammox biofilm demonstrated a robust capacity to achieve a high total nitrogen removal efficiency (TNRE) of 93 ± 5 % in MBBR and a total nitrogen conversion rate (TNCR) of 6.97 ± 1.30 mg N g⁻^1^ TSS d⁻^1^ in batch-scale under both-anoxic and combined aerobic/anoxic conditions. Additionally, the biofilm effectively eliminated PhACs present at both-high (0.8 mg L⁻^1^) and low (0.06 mg L⁻^1^) concentrations within a short time frame of 6 h batch tests. Higher initial PhAC concentrations showed greater removal efficiencies of them at 71.27 ± 6.71 % and 72.42 ± 7.92 % for CIP and NOR, respectively. Presence of a diverse bacterial community, with a significant proportion of *Candidatus Brocadia*, played a crucial role in maintaining the resilience and efficiency of the biofilm in antibiotic-rich environments. Findings also suggest that the anammox biofilm possesses a notable short-term resistance to combined antibiotic stress, which is crucial for its practical application in treating PhACs containing wastewater. Despite these positive outcomes, there are a few drawbacks to consider. The anammox process is more vulnerable to environmental factors like high PhAC concentration, low temperature, and pH, affecting the treatment stability and efficacy. Additionally, slow growth rate of anammox bacteria requires long start-up periods, making it challenging to implement the process in wastewater treatment facilities rapidly. Complex organic matter (PhACs) in real wastewater can also inhibit anammox activity, necessitating further research to optimize pre-treatment processes. The insights gained from this study lay the ground for further exploration and refinement of anammox processes in the context of PhACs' environmental biotechnology and wastewater management.

## CRediT authorship contribution statement

**Faysal-Al Mamun:** Writing – review & editing, Writing – original draft, Validation, Software, Methodology, Investigation, Formal analysis, Data curation, Conceptualization. **Rohit Kumar:** Writing – review & editing, Software, Formal analysis. **Kelvin Ugochukwu Anwuta:** Validation, Methodology, Investigation. **Sovik Das:** Writing – review & editing, Validation. **Madis Jaagura:** Methodology, Investigation. **Koit Herodes:** Writing – review & editing, Methodology, Investigation. **Tetyana Kyrpel:** Methodology, Investigation. **Agnieszka Fiszka Borzyszkowska:** Writing – review & editing, Validation. **Anna Zielińska-Jurek:** Writing – review & editing, Validation. **Zane Vincevica-Gaile:** Writing – review & editing. **Juris Burlakovs:** Writing – review & editing. **Andrey E. Krauklis:** Writing – review & editing, Validation. **Mohamad Nor Azra:** Validation. **Md Salauddin:** Writing – review & editing, Methodology. **Jiexi Zhong:** Writing – review & editing, Methodology. **Taavo Tenno:** Resources. **Kai Bester:** Writing – review & editing, Validation, Supervision. **Ivar Zekker:** Writing – review & editing, Validation, Supervision, Resources, Project administration, Funding acquisition, Formal analysis, Conceptualization.

## Data availability statement

Data will be made available on request.

## Declaration of competing interest

The authors declare that they have no known competing financial interests or personal relationships that could have appeared to influence the work reported in this paper.
