# Supplementary material for: How resistant is anammox biofilm against antibiotics: A special insight into anammox response towards fluoroquinolones
Source: Heliyon. 2024 Dec 18;11(1):e41339. doi: 10.1016/j.heliyon.2024.e41339 (PMC11729660; doi:10.1016/j.heliyon.2024.e41339)
Supplement: Multimedia component 1 [file mmc1.docx]

**Supplementary Material**

**How resistant is anammox biofilm against antibiotics: a special insight into anammox response towards Fluoroquinolones**

Faysal-Al Mamun^1^, Rohit Kumar^1^, Kelvin Ugochukwu Anwuta^1^, Sovik Das^2^, Madis Jaagura^3^, Koit Herodes^4^, Tetyana Kyrpel^4^, Agnieszka Fiszka Borzyszkowska^5^, Anna Zielińska-Jurek^6^, Zane Vincevica-Gaile^7^, Juris Burlakovs^8^, Andrey E. Krauklis^9^, Mohamad Nor Azra^10^, Md Salauddin ^11^, Jiexi Zhong^12^, Taavo Tenno^1^, Kai Bester^12,13*^, Ivar Zekker^1*^

*^1^Institute of Chemistry, University of Tartu,* *14a Ravila St., 50411 Tartu, Estonia.*

*^2^Indian Institute of Technology Delhi, Hauz Khas, New Delhi-110 016, India.*

*^3^Institute of Genomics, University of Tartu, Riia 23b, Tartu 51010, Estonia*

*^4^Institute of Chemistry. Chair of Analytics, University of Tartu, 14a Ravila St., 50411 Tartu, Estonia.*

*^5^Department of Environmental Toxicology, Faculty of Health Sciences, Medical University of Gdansk, Dębowa Str. 23A, 80-204 Gdansk, Poland.*

*^6^Department of Process Engineering and Chemical Technology Gdansk University of Technology Narutowicza 11/12, 80-233 Gdansk, Poland*

*^7^ Department of Environmental Science, University of Latvia, Jelgavas Street 1, LV-1004, Riga, Latvia.*

*^8^ Faculty of Civil and Mechanical Engineering, Riga Technical University, LV-1048 Riga, Latvia.*

*^9^ ASEMlab – Laboratory of Advanced and Sustainable Engineering Materials, Department of Manufacturing and Civil Engineering, NTNU – Norwegian University of Science and Technology, 2815 Gjøvik, Norway.*

*^10^Institute of Climate Adaptation and Marine Biotechnology, Universiti Malaysia Terengganu, 21030 Kuala Nerus, Terengganu, Malaysia.*

*^11^UCD Dooge Centre for Water Resources Research, School of Civil Engineering, University College Dublin, Ireland.*

*^12^Department of Environmental Science, Aarhus University, Frederiksborgvej 399, Roskilde 4000, Denmark.*

*^13^WATEC – Centre for Water Technology, Aarhus University, Ny Munkegade 120, Aarhus 8000, Denmark.*

*^*^Corresponding author: [Ivar.Zekker@ut.ee](mailto:Ivar.Zekker@ut.ee) (Ivar Zekker), [kb@envs.au.dk](mailto:kb@envs.au.dk) (Kai Bester)*

**Number of Figures: 5; Number of Tables: 1**

1. **Analyses of N-compounds**

**1.1. NO_2_^-^-N**

0.1-5 mL of the sample was added to a 25 mL volumetric flask and the flask was filled to the mark with deionized water. Then 0.5 mL of sulfanylamide (C_6_H_8_N_2_O_2_S) and 0.5 mL of diamine was added. After 10 minutes of reaction, the result was measured spectrophotometrically with a 10 mm cuvette on Hach Lange DR 2800 machine. The result was given in mg N / L and adjusted to the dilution of the sample.

**1.2. NH_4_^+^-N**

0.5-20mL of sample was added to a 25 mL graduated flask and the flask was ¾ filled with deionized water. Then 3 drops of mineral stabiliser and 2 drops of polyvinyl alcohol dispersing agent were added to the solution. Then, the flask was filled to the mark with deionized water and mixed using a flask cover. After that, 1 mL of Nessler reagent was added to the solution and a timer was set for 2 minutes, after which the sample was measured spectrophotometrically using an inch cuvette and a Hach Lange DR 2800 machine. The result was given in mg-N / L and adjusted to the dilution of the sample.

**1.3. NO_3_^-^-N**

1 mL of sample and 1 mL of sodium salicylate was evaporated on a water bath in ceramic evaporating dishes. After the evaporation, the sample was set aside until cooled to room temperature, after which 1 mL of sulfuric acid (H_2_SO_4_) was added to dissolve the dried sample. After waiting for 15 min and once the sample was completely dissolved, 8 mL of deionized water and 10 mL of NaOH + EDTA (sodium hydroxide & ethylenediaminetetraacetic acid) solution was added. Then, the sample was transferred to a volumetric flask with the size chosen according to the colour of the sample - the darker the yellow, the higher the nitrate concentration, and the greater dilution had to be made and the larger flask had to be used: size 25 mL, 50 mL or 100 mL. The flask was filled with deionized water to the mark and measured using the inch cuvette in the spectrophotometer Hach Lange DR 2800. The result was given in mg N / L and adjusted to the dilution of the sample.

**Tables**

**Table S1:** Summary of adsorption efficiency of PhACs compounds on the biofilm surface

| **Condition** | **CIP** | **NOR** | **OFL** |
| --- | --- | --- | --- |
| With slid particles | 436.4± 23.26 | 293.6 ± 27.52 | 116.6 ± 18.36 |
|  |  |  |  |
| After 5 min for precipitation | 376.6 ±17.73 | 257.6 ± 12.57 | 110.5 ± 14.25 |
| Adsorption % | 14% | 12% | 5% |

**Figures**


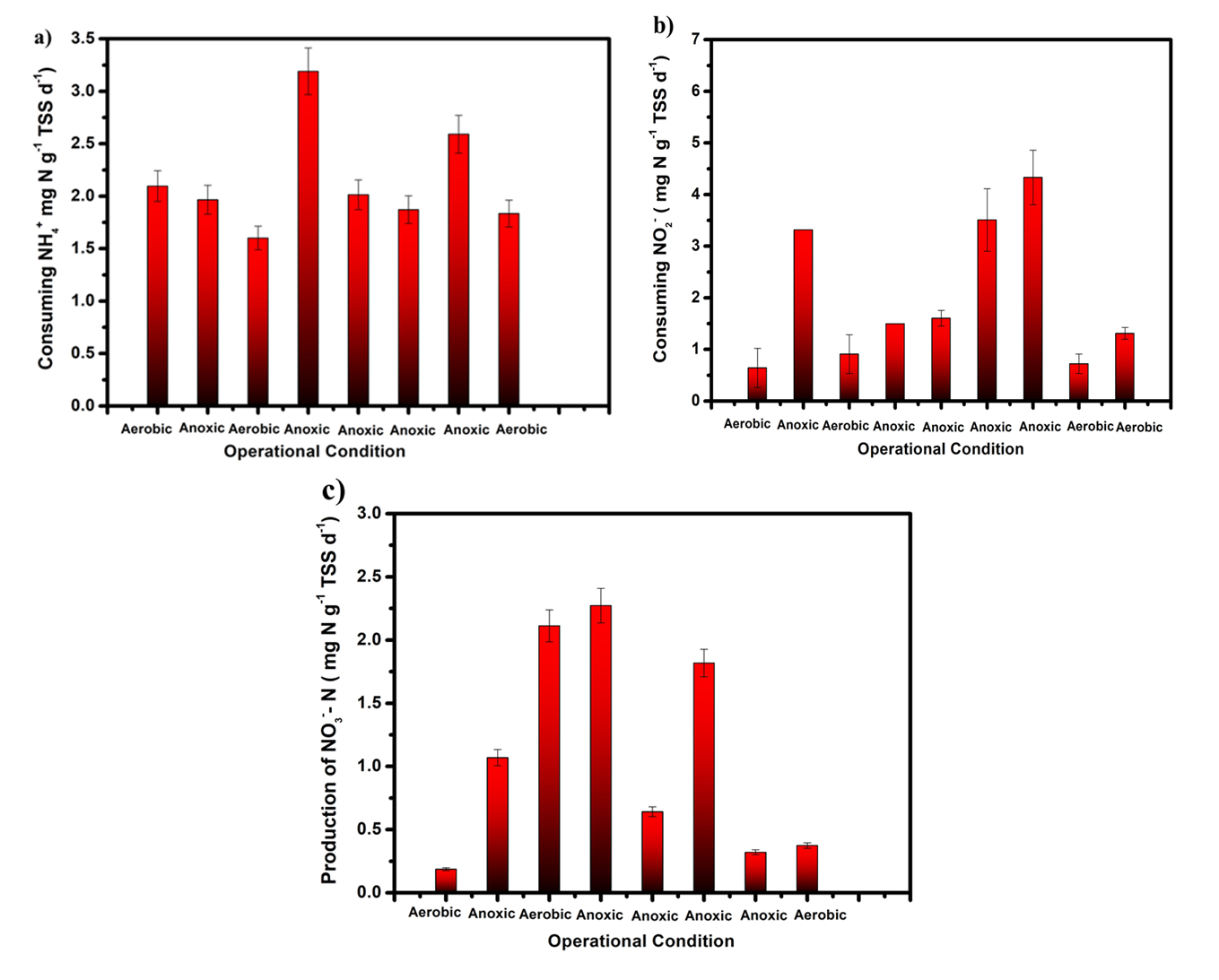


**Fig. S1**: Consumption and production rate estimated for NH_4_^+^-N, NO_2_^-^-N and NO_3_^-^-N species under various operational conditions.

**
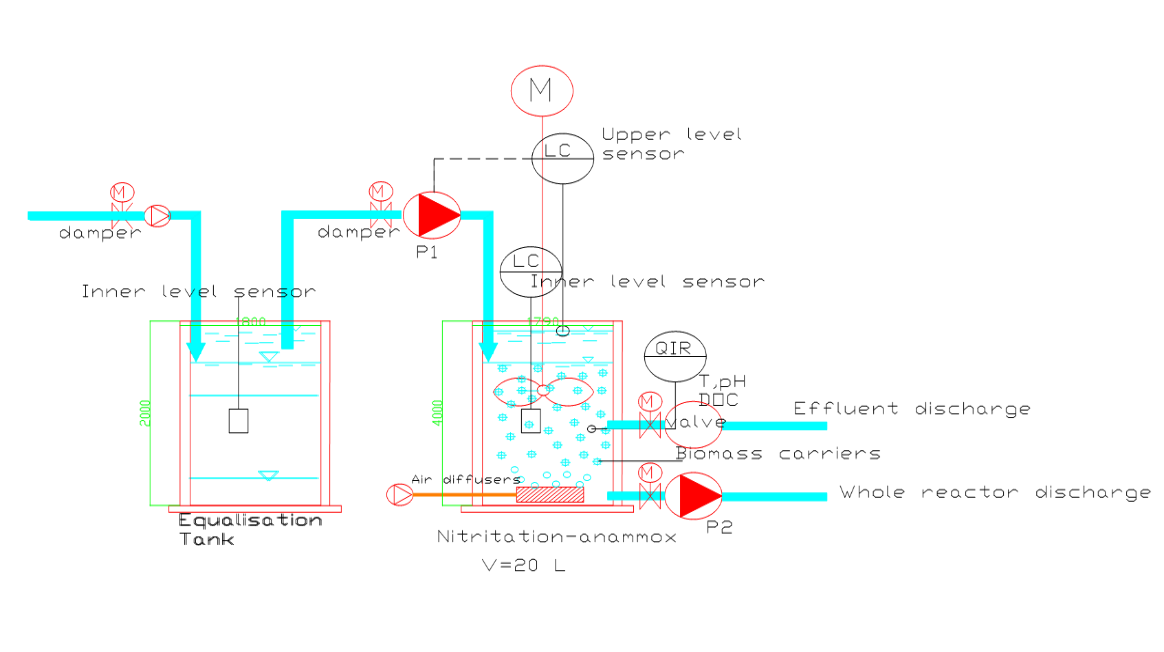
**

**Fig. S2:** Schematic representation of the MBBR setup used during this study.

**Fig. S3:** Change in pH of influent and effluent collected from the MBBR reactor


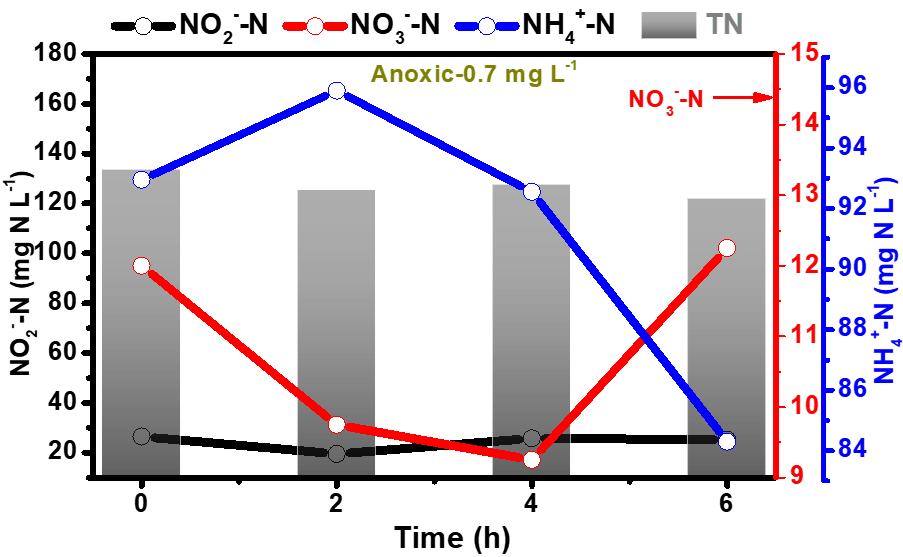


**Fig. S4:** Change in nitrogen species (NH_4_^+^-N, NO_2_^-^-N, and NO_3_^-^-N) concentration with time under anoxic conditions and in the presence of different PhACs inlet concentration of 0.7 mg L^-1^


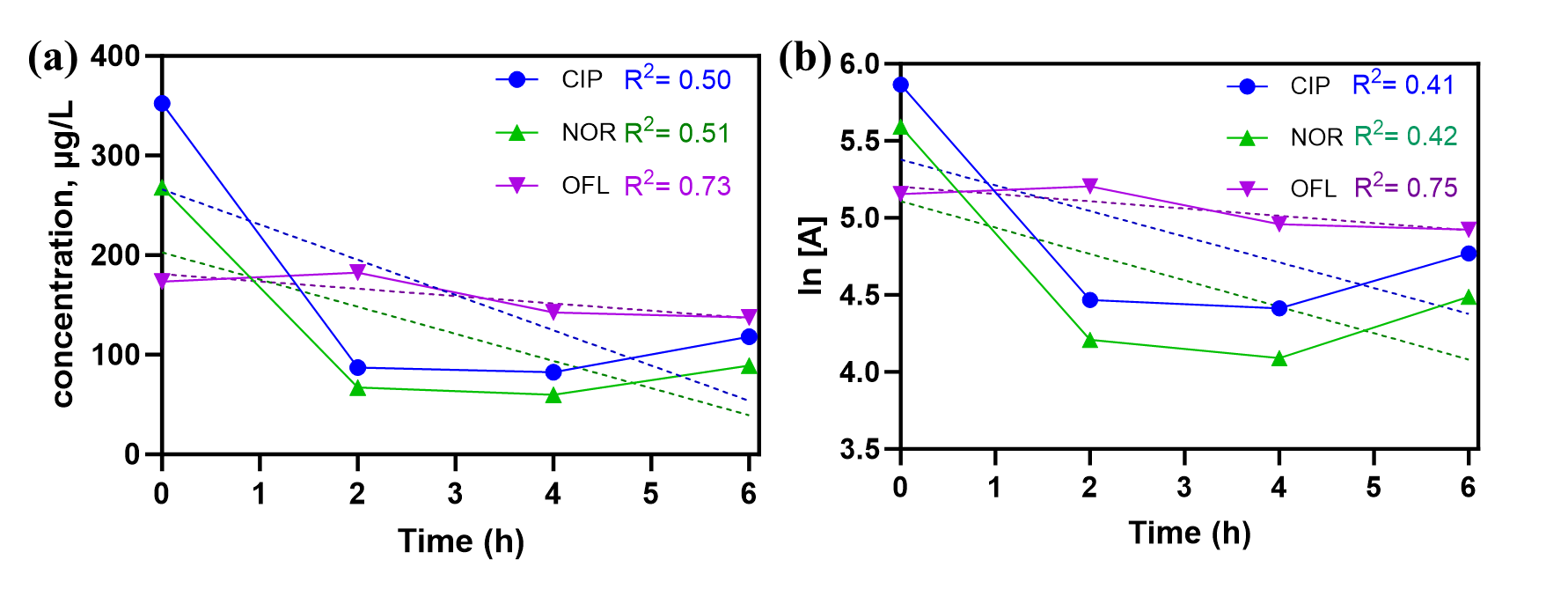


**Fig. S5:** (a) Zero-order and (b) First-order kinetic model fitting for PhACs compounds
